# Tracking interfacial single-molecule pH and binding dynamics via vibrational spectroscopy

**DOI:** 10.1126/sciadv.abg1790

**Published:** 2021-06-04

**Authors:** Junyang Huang, David-Benjamin Grys, Jack Griffiths, Bart de Nijs, Marlous Kamp, Qianqi Lin, Jeremy J. Baumberg

**Affiliations:** NanoPhotonics Centre, Cavendish Laboratory, Department of Physics, JJ Thompson Avenue, University of Cambridge, Cambridge CB3 0HE, UK.

## Abstract

Understanding single-molecule chemical dynamics of surface ligands is of critical importance to reveal their individual pathways and, hence, roles in catalysis, which ensemble measurements cannot see. Here, we use a cascaded nano-optics approach that provides sufficient enhancement to enable direct tracking of chemical trajectories of single surface-bound molecules via vibrational spectroscopy. Atomic protrusions are laser-induced within plasmonic nanojunctions to concentrate light to atomic length scales, optically isolating individual molecules. By stabilizing these atomic sites, we unveil single-molecule deprotonation and binding dynamics under ambient conditions. High-speed field-enhanced spectroscopy allows us to monitor chemical switching of a single carboxylic group between three discrete states. Combining this with theoretical calculation identifies reversible proton transfer dynamics (yielding effective single-molecule pH) and switching between molecule-metal coordination states, where the exact chemical pathway depends on the intitial protonation state. These findings open new domains to explore interfacial single-molecule mechanisms and optical manipulation of their reaction pathways.

## Teaser

Trapping light to atomic scales enables yoctoliter spectroscopy, unveiling individual molecular deprotonation events.

## INTRODUCTION

Ligand-nanoparticle surface interactions play key roles in catalysis and nanotechnology, from particle stabilization (*1*) to functionalization (*2*) for biosensing (*3*) and drug delivery applications (*4*). In a number of recent studies, ligands have been engineered to promote the efficiency and selectivity of heterogeneous catalysis on metal nanocrystals (*5*–*9*) despite the previous consensus that all the surface agents are detrimental to this process through blocking of catalytically active surface sites. Ligands with termination groups, such as carboxylates, can bind to metal surface atoms via a variety of possible coordination modes (*5*), enabling steric (*6*, *7*) and electronic (*8*) modification of the catalytic efficiency of metal nanoparticles and accessing the potential of chemoselectivity (*9*). In addition, proton activities at the metal interface surface play a central role in ligand tethering (*10*) as well as catalytic (*11*–*13*) and electrochemical processes (*14*), yet the interfacial pH at molecular length scales markedly varies from the bulk environment and is challenging to track (*15*).

Typical characterization methods, including electrochemistry (*16*), nuclear magnetic resonance (*17*), infrared (*18*), and x-ray photoelectron spectroscopies (*1*), measure an ensemble average of ligands in chemical equilibrium. To provide dynamic information at the single ligand level, which is required for the design of next-generation functional nanomaterials (*19*), single-molecule optical spectroscopy techniques can now be used. While single-molecule fluorescence spectroscopy is able to resolve real-time catalytic (*20*–*22*) and tautomerization (*23*) processes at metal interfaces, the lack of direct structural information obscures the precise chemical transformations taking place. Surface-enhanced Raman spectroscopy (SERS) is an inelastic scattering method that provides structural information on unlabeled molecules near the surface of noble metal nanostructures, without relying on molecules to fluoresce (that bleach over time). It can therefore serve as a powerful technique for in situ in operando tracking of interfacial chemical reactions (*24*–*27*). Recent advances in single-molecule SERS and tip-enhanced Raman spectroscopy (TERS) are thus opening up these new possibilities for single-molecule real-time vibrational characterization (*28*–*32*).

Nanoscale gaps between plasmonic metal surfaces confine optical fields far below the free-space diffraction limit to effective mode volumes on the order of 10^2^ nm^3^, enabling access to plasmon-enhanced spectroscopy at the single-molecule level (*33*). Laser-induced atomic surface protrusions created within these optical nanocavities offer a further level of field localization via an atomic-scale lightning rod effect (*34*), resulting in an optical “picocavity” of yoctoliter effective volume (<1 nm^3^) centered around a single surface adatom (*35*). This picocavity spatially isolates a single molecule from the nanocavity hotspot within a region of intense field strength, which provides additional SERS enhancement with respect to the background nanocavity field (∣*E*_pico_/*E*_nano_∣^4^ ≃ 80). Extreme field gradients within this picocavity can further enhance certain vibrational modes including those that are typically dark, depending on the position and orientation of the molecule with respect to the nearby adatom (*36*). This spectrally spotlights the molecule from the ensemble while overcoming the typically low single-molecule SERS cross sections and rendering them experimentally resolvable in both cryogenic and ambient conditions (*34*, *36*, *37*).

Here, we use long-lived picocavities within a cascaded SERS substrate to enable in situ optical tracking of individual 3–mercaptopropionic acid (MPA) molecules undergoing structural transformations at the surface of a gold nanoparticle (AuNP) under ambient conditions. This reveals distinct three-level chemical oscillations of the carboxylic group. Combining this with theoretical calculations, we identify reversible proton transfer dynamics as well as subsecond switching between single-molecule coordination states, where the chemical pathway observed varies depending on the initial protonation state. Essentially, this quantifies pH at the single-molecule level. Exemplified here is a robust strategy to probe the mechanisms of such single-molecule interfacial reactions.

## RESULTS AND DISCUSSION

### MPA nanojunction

**Fig. 1:**
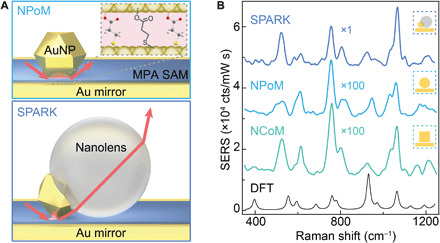
Plasmonic cavity for MPA SERS sensing. (**A**) Self-assembled MPA monolayer (blue) sandwiched in NPoM (top) and SPARK (bottom) geometries. Red arrows indicate optical in- and out-coupling angles. (**B**) Time-averaged SERS spectra of the MPA monolayer collected from SPARK, NPoM, and NCoM nanocavities with, respectively, 20-μW, 500-μW, and 1.2-mW optical excitation at 633 nm (300 s integration time), together with simulated Raman spectrum for monodentate MPA using polarized DFT calculations.

We sandwich a self-assembled monolayer (SAM) of 3-mercaptopropionic acid (MPA) molecules (*38*, *39*) between sparsely deposited 80-nm AuNPs and a template-stripped gold surface (the Au mirror). Upon high-angle excitation of these nanoparticle-on-mirror (NPoM) (*33*) nanostructures, plasmonic coupling between the nanoparticle and its mirror-image charges results in a highly reproducible SERS hotspot inside the 0.8-nm-thick gap defined by the MPA monolayer (Fig. 1A and fig. S1) (*40*). Even with the enhancement provided by this nanocavity, the low SERS cross section of MPA limits the speed of observations to tens of seconds (Fig. 1B, light blue). Similar signals are achieved using a nanocube-on-mirror (NCoM) (80 nm) geometry (Fig. 1B, green) (*40*).

Recent development of Super-efficient Plasmonic nanoArchitectures for Raman Kinetics (SPARKs) (*41*) substantially improves the SERS efficiency of the NPoM structure, providing enhancement factors exceeding 10^11^ without degrading the molecular layer and thus enabling faster spectral collection. Such metallo-dielectric hybrid nanostructures consist of a AuNP (80 nm) partially embedded in an organosilica nanolens (refractive index *n* = 1.5) (see fig. S2 for TEM and SEM characterization). When deposited on a SAM-coated Au film, the metallo-dielectric particles form this SPARK geometry, enhancing optical in-coupling and out-coupling efficiencies through the combined contribution of near-field nanolensing, symmetry breaking, resonant whispering gallery mode reexcitation of the plasmonic modes, and light concentration through the down-facing sharp contact of the AuNP (*41*). Experimentally, for an MPA monolayer with 785-nm excitation, SPARK nanocavities yield approximately 100 times higher SERS signals compared to NPoM and NCoM nanocavities (Fig. 1B), despite probing fewer molecules (*41*). Such increased SERS sensitivity is critical to probe chemical processes at higher time resolutions and uses weak (nonperturbing) optical powers.

Upon SAM formation at pH 4, the MPA carboxylate group is left mostly protonated (p*K*_a_ = 4.3) (*42*) while the thiol group anchors the molecule on the gold mirror through Au─S bonding (fig. S3). After AuNP deposition to form either NPoM, NCoM, or SPARK nanocavities, the MPA molecules within the nanogaps can exist in four chemically distinct coordination states: protonated (COOH), deprotonated (COO^−^), monodentate (η^1^-COO^−^), and bidentate bridge [μ_2_-(η^2^-COO^−^)] coordination to the upper AuNP facet (fig. S4B). Consistent time-averaged nanocavity SERS spectra (>300 s) are observed using all nanoconstructs (Fig. 1B). Careful comparison to spectra simulated using density functional theory (DFT) suggests that the population of MPA molecules in the nanocavity is dominated by the monodentate coordination state (Fig. 1B, black; see DFT of other states in fig. S4C) (*40*).

### Real-time reversible single-proton transfer dynamics

Picocavities can be generated stochastically within these nanocavity constructs via laser irradiation. To monitor the time-resolved spectral evolution of these picocavity events within a SPARK structure, SERS spectra are continuously recorded during laser irradiation at 633 nm (see Materials and Methods). The combined signal amplification from both the SPARK and picocavity allows a reduction in spectrum integration time to 50 ms (with sub–100-μW irradiation) to interrogate the dynamics of single MPA molecules on a much faster time scale. To date, previous picocavities formed at room temperature have not been stable for more than a few seconds under optical irradiation (*36*, *37*). It has been reported that the energy barrier to both generate adatoms and return them to the bulk lattice is strongly dependent on the molecules at the metal surface (*37*). In marked contrast to previously studied biphenyl molecules, picocavities formed in the presence of MPA are extraordinarily long-lived at room temperature and equivalent laser powers, with lifetimes regularly exceeding minutes. Such stability, attributed to the strong molecule-metal interaction, uniquely enables single-molecule tracking for an extended period of time. The lifetime and stability of the picocavity are known to be sensitive to the incident laser power after its formation (fig. S5). To ensure minimal optical disturbance of the picocavities formed, 50-μW optical excitation is used, balancing stability against an acceptable signal-to-noise ratio.

**Fig. 2:**
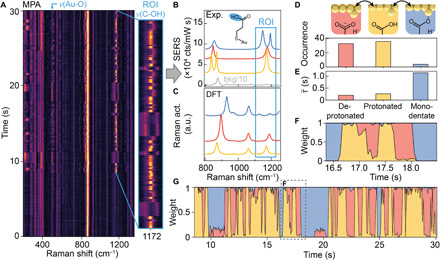
Single-molecule chemical dynamics of MPA. (**A**) SPARK SERS time series showing single-molecule dynamics of MPA, with 50-ms integration time per spectrum. (**B**) Pure state experimental spectra (blue, red, and yellow) and background (gray) extracted from SERS time scan. (**C**) DFT-calculated spectra for Au-S anchored MPA molecule in a protonated (yellow), deprotonated (red), and monodentately coordinated (blue) configuration. Region of interest (ROI) highlights vibrations corresponding to a mixture of ν(C-OH), ν(C-O)-Au stretching, and ω(CH_2_) wagging modes in different chemical states of (D). (**D**) Schematics and occurrence of the three MPA picocavity states (top) during the 22-s highlighted time window in (A), with (**E**) average dwell time of the three states. (**F** and **G**) Time evolution of the extracted fractional weights for the three pure states, showing dynamic switching behavior, with (F) showing a magnified time window from (G). a.u., arbitrary units.

During the progress of a picocavity event, MPA SERS lines switch digitally between three discrete spectral states (Fig. 2A). This repetitive digital switching phenomenon contrasts with previous continuous spectral wandering caused by angstrom-level variations in adatom-molecule separation (*43*). We attribute such spectral features to a single molecule because adatoms can sterically interact with only one molecule and would give separate (split) lines for more molecules, and their light fields reach out only 20 pm (*36*). As MPA spends time in multiple states within each integration time, we decompose the experimental spectra into linear combinations of the three “basis spectra” and a time-independent background (Fig. 2B and figs. S6 and S7). These states are distinguished within the region of interest (ROI) [1120 to 1220 cm^−1^ around ν(C-OH) stretching region] by peaks at 1171 cm^−1^, 1175 cm^−1^, or a doublet at 1147/1193 cm^−1^ (Fig. 2B, red, yellow, or blue, respectively). The relative positions of these modes are well matched with the DFT-calculated modes of protonated MPA, deprotonated MPA, and MPA monodentate coordination to a gold atom, respectively (Fig. 2, C and D).

The spectral shift upon MPA deprotonation results from a redistribution of electron density within the carboxylate group and a subsequent increase in the C_3_-C_4_ vibrational energy (Fig. 2D, top). Coordination bond formation between a carboxylate oxygen atom and a heavy gold adatom breaks the symmetry between the two oxygen atoms, leading to the observed peak splitting in the monodentate state (blue) accompanied by an intensified ν(Au-O) line at 500 cm^−1^. The relative peak shift of the C_2_-C_3_ stretching mode (around 850 cm^−1^) is also in agreement with the DFT calculation, as well as literature spectroscopic studies on carboxylate groups (*44*). The exact positions and their relative intensity in each picocavity are not only sensitive to the local lattice environment (fig. S4, E and F) but also strongly affected by both the relative separation and the angular alignment between the molecule and adatom (*43*, *45*). The consistent relative changes in the spectral features allow us to assign the basis spectra to these three distinct chemical states: protonated, deprotonated, and monodentate MPA.

Classification of each spectrum yields further statistics of the chemical dynamics. A significantly lower occurrence is found for the monodentate state, while equally frequent appearances are recorded for the protonated and deprotonated states (Fig. 2D). However, once the monodentate coordination is formed, the molecule spends the longest time on average in this state. On the other hand, the mean dwell time for both protonated and deprotonated states is short (<0.3 s, Fig. 2E). This difference in the dwell times implies that the energy barrier between protonated and deprotonated states is small compared with the energy needed for the molecule to unbind from the gold surface, reflecting that O─H binding is weaker than the O─Au coordination bond. The time-evolving normalized weights of the three basis states reveal the digital chemical hopping behavior of a single molecule on a subsecond time scale (Fig. 2G). Within the highlighted time window (Fig. 2F), the MPA molecule first retracts the Au-carboxylate coordination by recapping the carboxylate group with a proton. The molecule then fluctuates between the protonated and deprotonated states before it returns to the monodentate coordination with Au. Fractional weights between two states suggest that the chemical switching under a thermally driven dynamic equilibrium is faster than the spectral integration time (limited by the photon yield), with their relative energy barriers dictating the fractional weight observed.

### Tracking chemical switching among molecule-metal coordination states

**Fig. 3:**
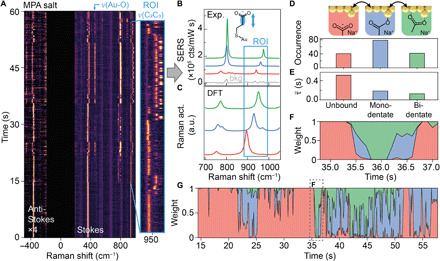
Single-molecule coordination dynamics of MPA salt. (**A**) SPARK SERS time series showing single molecular dynamics of MPA salt, with 100-ms integration time. (**B**) Pure state spectra (green, blue, and red) and background (gray) extracted from the SERS time scan. (**C**) DFT-calculated spectra for an MPA molecule unbound (green), monodentately coordinated (blue), and bidentately coordinated (green) to Au. ROI highlights vibrations corresponding to ν(C_2_C_3_) in different chemical states. (**D**) Schematics and occurrence of the three coordination states of MPA (top) in a picocavity during the 43-s highlighted time period in (A). (**E**) Average dwell time of the three coordination modes. (**F**) Short segment of the time evolution of the extracted fractional weights for the three pure coordination states, capturing sequential hopping among them. (**G**) Full time evolution of a single MPA molecule hopping between three coordination states, with (F) showing a magnified time window from (G).

Producing the SAM using a sodium salt form of MPA (pH 7), instead of MPA at pH 4, alters the local chemical environment in the nanogap by drastically reducing the number of available protons and associating heavier sodium ions instead. In this system, picocavity SERS spectra retain digital switching behavior among three chemically distinctive states (Fig. 3A). As expected, the protonated state is no longer observed (Fig. 3B). Pairing the basis states with DFT-calculated reference spectra (Fig. 3C) shows the closest match for deprotonated, monodentate, and, now, also a bidentate binding, indicating the formation of a Au─O coordination bond on both carboxylic oxygen atoms. The formation of Au─O bonds under both monodentate and bidentate coordination is evidenced by the appearance of a SERS line at 460 cm^−1^ together with a simultaneous blue shift of the ν(Au-S) from 360 to 380 cm^−1^. The monodentate coordination state is found to be the most frequently visited state, occurring twice as often as the unbound state and the bidentate state (Fig. 3D), while a significantly longer dwell time is recorded for the unbound state. An exemplar 2-s time window (Fig. 3F) shows the stepwise transition from the unbound state to monodentate then to bidentate and vice versa. This is consistent throughout: For the molecule to form bidentate coordination, the monodentate state is always visited as an intermediate step, evidencing how the two coordination bonds on the carboxylate group form and break in a stepwise fashion.

During irradiation, changes in the average occupation time of the three states can be observed (Fig. 3G). In this example, during the first period (15 to 35 s), the molecule resides mainly in the unbound state, while the bidentate state is barely registered. By contrast, from 35 s onward, the molecule spends most of its time in one of the two coordination modes and significantly less time in the unbound state. As we will show, such changes evidence evolution of the local chemical landscape.

### Statistical analyses

**Fig. 4:**
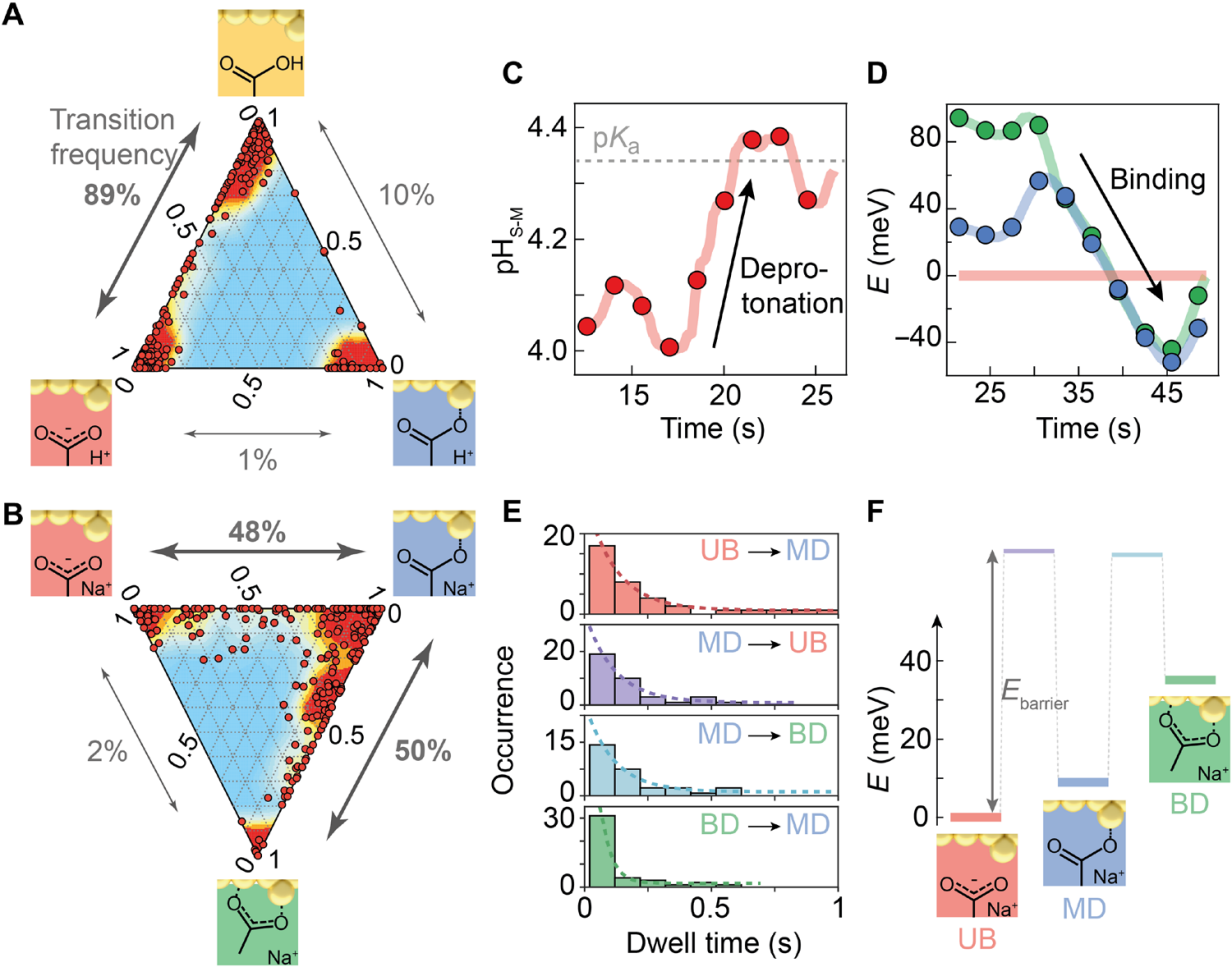
Evolving chemical landscape for a single molecule. (**A** and **B**) Distribution and contour map of picocavities from (A) MPA and (B) MPA-sodium-salt, across the chemical space, in ternary representations, where each data point represents the distribution in states for a picocavity spectrum. The frequency of hopping between states is marked in gray along each axis. (**C**) Extracted effective single-molecule pH fluctuation showing deprotonation is increasingly favored in time (p*K*_a_ = 4.3). (**D**) Variation of equilibrium energies of the monodentate (blue) and bidentate (green) states versus the unbound state extracted from the transition kinetics of the MPA salt. (**E**) Distributions of dwell times in the unbound (UB), monodentate (MD), and bidentate (BD) states of an MPA salt picocavity before hopping to a neighbor state, with fitted exponential decay constants of τ_UB→MD_ = 118 ± 2 ms, τ_MD→UB_ = 120 ± 20 ms, τ_MD→BD_ = 110 ± 20 ms, and τ_BD→MD_ = 41 ± 7 ms. (**F**) Relative energy landscape of a deprotonated MPA molecule in a picocavity, extracted using the average occupancy and dwell time decay rates, where transition states are marked with purple and light blue (the barrier height remains undetermined).

With the molecular dynamics characterized for both the MPA and MPA-salt SAMs, we further analyze and compare their difference in chemical kinetics to provide powerful insights into the atomic-scale behavior of the molecule. We note that similar features are seen in all scans (fig. S8). With the acidic MPA SAM, the transition between protonated and deprotonated states dominates the chemical switching kinetics with 89% of registered switching events (Fig. 4A). Switching to the monodentate state is significantly rarer and predominantly occurs from the deprotonated state, accounting for 10% of transitions. Switching from the protonated state directly to the monodentate state accounts for only 1% of transitions, suggesting that the presence of the capping proton plays an important role in the formation and dissociation of the metal coordination bond. The overall rarity of the monodentate state and the observed longer lifetime for this state suggest that it is energetically less favorable and has a higher activation barrier.

While MPA is a weak acid that partially dissociates in solution during SAM preparation, MPA salt fully dissociates all the sodium ions in solution, exposing carboxylate groups to make them more likely to bind to the metal. The dynamics observed indeed shows that for the MPA-salt SAM, the transition from deprotonated to monodentate now accounts for 48% of observed switches (Fig. 4B). The transition from monodentate to bidentate now accounts for 50% of the state changes, with the transition from deprotonated to bidentate accounting for a small fraction (2%). The distribution of picocavities (Fig. 4B) for the MPA-salt SAM is more widely scattered and attributed to the longer integration times used, which increases the probability of capturing more than one state in a single spectrum.

Although the stochastic reservoirs of the system are not characterized, sufficient switching events are registered in a time scan to support the assumption that the molecule is thermalized during the total spectral acquisition time. The picocavity vibrational frequencies for both the protonated and deprotonated states are consistent throughout the scan, indicating that the electronic environment of the MPA molecule is stable and therefore supporting the assumption that the p*K*_a_ of the molecule is not significantly perturbed within the picocavity. From the time-averaged occupation probability of the protonation states, we then evaluate an effective single-molecule pH value for a single MPA molecule, pH_S−M_, using a modified version of the Henderson-Hasselbalch equation pH_S−M_ = p*K*a + log_10_(*P*_A^−^_/*P*_HA_), where *P*_A^−^_ and *P*_HA_ are the occupation probabilities of the deprotonated and protonated states, respectively. The real-time pH fluctuation shows that the molecule is more stable in the protonated state in the first 20 s (Fig. 4C), while occupation of the deprotonated state can be thermally driven (by coupling to the reservoir of phonons in the metal and molecule). A shift in this chemical equilibrium is observed around 20 s, whence the pH increases toward the p*K*_a_ value. However, comparing different SERS time scans, this fluctuation of single-molecule pH does not happen always the same way (fig. S8). The relative energy between chemical states can also be retrieved from the relation *E*_∆*AB*_ = −*k*_B_*T* ln(*P_A_*/*P_B_*), where *P* is the probability of occupying a state, *k*_B_ is the Boltzmann constant, and *T* is the molecular temperature, which is estimated to be 305 K, using the ratio between anti-Stokes and Stokes scattering intensities (*46*). Extracting the energies from the chemical kinetics of MPA salt (Fig. 4D) reveals a tilt in chemical equilibrium during continuous laser irradiation. The energies of the three chemical states are stable and well separated in the first 30 s. Later, the bidentate state becomes equal energetically to the monodentate state, while both binding configurations evolve to be more energetically favorable compared with the unbound state. This suggests that the observed modification of single-molecule chemical equilibria is light-driven. Without clear evidence of optical heating seen, we thus suggest that optical forces at the atomic scale can also play a role, which may thus be critical for photochemistry. This direction is of considerable interest for future work.

The dwell times of the molecule in the three states obtained from the MPA-salt picocavity follow an exponential distribution (Fig. 4E), where the rate constants (1/τ) are associated with the energy barrier (*E*_B_) for the corresponding chemical transitions through 1/τ = ν exp(−*E*_B_/*k*_B_*T*). Without assuming a value for the molecular attempt frequency (ν), the ratio between the rate constants directly yields a small energy difference between the unbound-monodentate (purple) and bidentate-monodentate (light blue) transition states: *E*_MD→UB_ − *E*_MD→BD_ = ln(τ_MD→UB_/τ_MD→BD_)*k*_B_*T* = 1 ± 6 meV. This concurs with the fact that a similar number of hopping events are observed from the monodentate state to the unbound and bidentate states. Combining the relative barrier heights and energy levels from the average probability of occupation of the three states allows us to reconstruct a limited chemical energy landscape at the single-molecule level (Fig. 4F).

Although forming bidentate coordination is expected to lower the energy of the MPA molecule under standard conditions, it appears here to be the most energetic state. We suggest that the higher energies of the binding states arise from chloride anions initially bound on the gold surface (*47*). Surface chlorides have been shown previously to prevent citrate carboxylate from bidentate binding on AuNP surfaces (*44*). Furthermore, the limited degrees of freedom in the tightly confined geometry of the picocavity influence the energies of the binding states, depending on the relative distance and orientation angle between the adatom and the carboxylate group. In addition, we confirm that similar chemical switching dynamics is also observed using longer integration time measurements on the weaker SERS NPoM geometry with MPA molecules (fig. S9), as well as for two other carboxylic molecules: mercaptoacetic acid and 4-mercaptobenzoic acid (fig. S10). These observations confirm that the observed switching phenomenon between chemical states on Au is a general property of the carboxylate group and independent of the specific plasmonic structure.

In summary, we demonstrate how optically generated long-lived picocavities can reveal the dynamic structural transformation of reversible deprotonation and binding reactions at the single-molecule level under ambient conditions. The reliably high-efficiency SERS provided by the SPARK architectures now allows vibrational transformations to be resolved with 50-ms integration times for individual single MPA molecules. From the resulting three-level switching observed in the SERS kinetics, we identify proton transitions and fast oscillations between carboxylate surface coordination states. This explores real-time single proton dissociation dynamics for single molecules bound to metals. Stochastically fluctuating temporal trajectories reveal reaction pathways of the chemical states. Our data show that the chemical equilibrium and the effective pH of the MPA molecule can be shifted as a result of optical irradiation, which demonstrates the potential for optical tuning of the energy barriers between chemical states of the surface-bound molecules. This platform offers promising perspectives to explore interfacial single-molecule dynamics as well as light-matter interactions at many molecule-metal junctions. It thus affects the understanding and design of surface functionalization in a wide range of nanotechnology and photochemistry applications.

## MATERIALS AND METHODS

### SPARK sample preparation

The gold-organosilica heterodimers are prepared by nucleation and growth of (3-mercaptopropyl)trimethoxysilane (MPTMS) (Sigma-Aldrich) using AuNPs (Sigma-Aldrich) as seeds. The particle sizes are controlled as a function of MPTMS concentration, and the MPTMS wetting angle relative to the AuNP surface is obtained to be around 50°. Detailed synthetic processes are reported by Kamp *et al.* (*41*) Characterization using TEM micrographs shows that the gold-organosilica heterodimers used in this study have a AuNP diameter of 80 nm and a SiO_2_ diameter of 200 nm. To form the SPARK geometry for SERS measurement, the gold-organosilica heterodimers are deposited onto template-stripped gold coated with a SAM of MPA forming the SPARK geometry.

To form the SAM, 1 mM MPA or MPA sodium salt solutions are prepared and their pH values are determined using a pH probe (SciQuip-8008). Template-stripped gold (TSG) substrates are immersed in MPA solution for 20 hours to allow the SAM to saturate. The samples are then rinsed with deionized water and dried with nitrogen. For NPoM samples, AuNPs are deposited by dropcasting 80 μl of AuNP dispersion (BBI Solutions, 80 nm diameter, OD1, citrate-buffered) onto an MPA-coated TSG mirror. After 30 s of deposition, excess AuNPs are rinsed off with deionized water and the sample is dried with nitrogen. To deposit SPARKs, a 60- to 100-μl ethanolic suspension of gold-organosilica heterodimers is placed onto the MPA decorated TSG substrate for 10 min to form the SPARK geometry, before rinsing with ethanol and drying with nitrogen.

### SERS measurements

SERS measurements are performed using a modified Olympus BX51 microscope in reflective dark-field geometry. SPARK and NPoM samples are excited with either a 633- or a 785-nm single-frequency continuous-wave diode laser (Integrated Optics). The scattered light is collected using a ×100 Zeiss objective (0.9 numerical aperture) and filtered with two notch filters before the signals are coupled through an Andor Shamrock 303i spectrograph and recorded on an Andor Newton EMCCD. On each SPARK geometry, multiple sets of 600 SERS spectra are taken in quick succession using an integration time of 50 or 100 ms.

### DFT calculations

DFT calculations are carried out using the Gaussian 09 (Rev. E.01) ab initio software suite (*48*). For the geometry optimization and frequency calculation, the hybrid functional PBE0 (PBE1PBE) in conjunction with the 6-311++G(d,p) and Los Alamos ECP double-ζ (LANL2DZ) basis sets are used. Empirical dispersion correction according to the model by Grimme with Becke-Johnson damping (GD3BJ) is included (*49*). Calculations are performed for both the gas phase and also including solvation effects using the polarizable continuum model. The orientation-averaged Raman activities were recalculated to account for polarization effects in the nanogap. This is achieved by extracting the polarizability derivatives from the Gaussian output (*50*).

## SUPPLEMENTARY MATERIALS

Supplementary material for this article is available at http://advances.sciencemag.org/cgi/content/full/7/23/eabg1790/DC1
